# Reconstructing the history of a fragmented and heavily exploited red deer population using ancient and contemporary DNA

**DOI:** 10.1186/1471-2148-12-191

**Published:** 2012-09-26

**Authors:** Jørgen Rosvold, Knut H Røed, Anne Karin Hufthammer, Reidar Andersen, Hans K Stenøien

**Affiliations:** 1 Section of Natural History, Museum of Natural History and Archaeology, Norwegian University of Science and Technology, N-7491, Trondheim, Norway; 2 Department of Basic Sciences and Aquatic Medicine, Norwegian School of Veterinary Science, Box 8146 Dep, N-0033, Oslo, Norway; 3 The Natural History Collections, Bergen Museum, University of Bergen, Box 7800, 5020, Bergen, Norway; 4 The Norwegian Nature Inspectorate, Box 5672, Sluppen, NO-7485, Trondheim, Norway

**Keywords:** Ancient DNA, Habitat fragmentation, Harvesting, Mitochondrial DNA, Genetic diversity, Translocation, *Cervus elaphus*

## Abstract

**Background:**

Red deer (*Cervus elaphus*) have been an important human resource for millennia, experiencing intensive human influence through habitat alterations, hunting and translocation of animals. In this study we investigate a time series of ancient and contemporary DNA from Norwegian red deer spanning about 7,000 years. Our main aim was to investigate how increasing agricultural land use, hunting pressure and possibly human mediated translocation of animals have affected the genetic diversity on a long-term scale.

**Results:**

We obtained mtDNA (D-loop) sequences from 73 ancient specimens. These show higher genetic diversity in ancient compared to extant samples, with the highest diversity preceding the onset of agricultural intensification in the Early Iron Age. Using standard diversity indices, Bayesian skyline plot and approximate Bayesian computation, we detected a population reduction which was more prolonged than, but not as severe as, historic documents indicate. There are signs of substantial changes in haplotype frequencies primarily due to loss of haplotypes through genetic drift. There is no indication of human mediated translocations into the Norwegian population. All the Norwegian sequences show a western European origin, from which the Norwegian lineage diverged approximately 15,000 years ago.

**Conclusions:**

Our results provide direct insight into the effects of increasing habitat fragmentation and human hunting pressure on genetic diversity and structure of red deer populations. They also shed light on the northward post-glacial colonisation process of red deer in Europe and suggest increased precision in inferring past demographic events when including both ancient and contemporary DNA.

## Background

Humans have a major impact on the evolution and survival of other life forms [1]. Our activities may modify the genetic diversity and structure of other species through e.g. heavy harvesting, habitat fragmentation and translocation of populations [2,3], often reducing their fitness and future adaptive potential [4,5]. Red deer (*Cervus elaphus*) are one of the most common and widespread of European ungulates today [6]. However, due to heavy hunting and habitat alterations, many populations were severely reduced in numbers in previous centuries. Several populations were driven to extinction or confined to scattered and isolated refugia from which they have later expanded [6]. Even today the widespread distribution is often patchy and fragmented, a trend that is increasing in some areas as a result of habitat loss and overhunting [7]. Red deer have also been subjected to poorly documented human mediated translocations of animals, often over large distances, leading to mixing with or substitution of the indigenous population [6]. Such translocations could potentially disrupt long-term local adaptations of populations [8].

Norwegian red deer represents the end point of the northward post-glacial colonisation of the western European red deer lineage [9] and relatively low genetic diversity has been found in contemporary populations [10,11]. Traditionally it was described as a separate subspecies (*C. e. atlanticus*), but this is not supported in more recent analyses based on skull morphology [12] or molecular phylogenetics [13,14]. Norwegian deer are, nevertheless, differentiated both morphologically and genetically from Swedish and Danish populations and seem more closely related to Scottish red deer [10,15]. This differentiation among Scandinavian red deer has been explained either by post-glacial separation and adaptations to different environments, or alternatively, that they originate from different source populations [15,16]. Explanations for the latter hypothesis suggest two waves of post-glacial immigration to northern Europe, with the first wave dominating the Norwegian population, and a later human mediated translocation of animals possibly by Vikings [10,15,17]. There are numerous findings of prehistoric red deer bones in Norway and, according to older documents e.g. [18], they were widespread and numerous in the 16^th^ century AD before rapidly declining in numbers, allegedly as an effect of increased hunting pressure and wolf predation. A similar decline was reported in Sweden [19] and in the 19^th^ century the Scandinavian red deer had been reduced to a few isolated locations, estimated to consist of only a few hundred animals in total [20]. Following strict hunting regulations, an almost exponential growth took place during the last century and the current estimated Norwegian census size is well above 100,000 individuals, mainly distributed along the west coast of the Scandes mountains [17].

In this study we analyse contemporary and ancient genetic diversity in Norwegian red deer in a time-series of mitochondrial DNA (mtDNA) sequences spanning the last 7,000 years. Our aim is to estimate the timing, pattern and magnitude of the historic population decline using different methods to detect past demographic changes. In addition, we explore the relationship to other European populations and the possibility of human translocation of red deer into Norway. We analyse contemporary DNA together with ancient DNA (aDNA) using Bayesian methods and investigate whether including aDNA significantly changes the outcome of our analyses. Our results provide direct insights into the effects of increasing agricultural land use and human hunting pressure on genetic diversity and structure on a long timescale, and shed light on the northward post-glacial colonization process of red deer in Europe.

## Methods

### Sampling and dating

**Figure 1:**
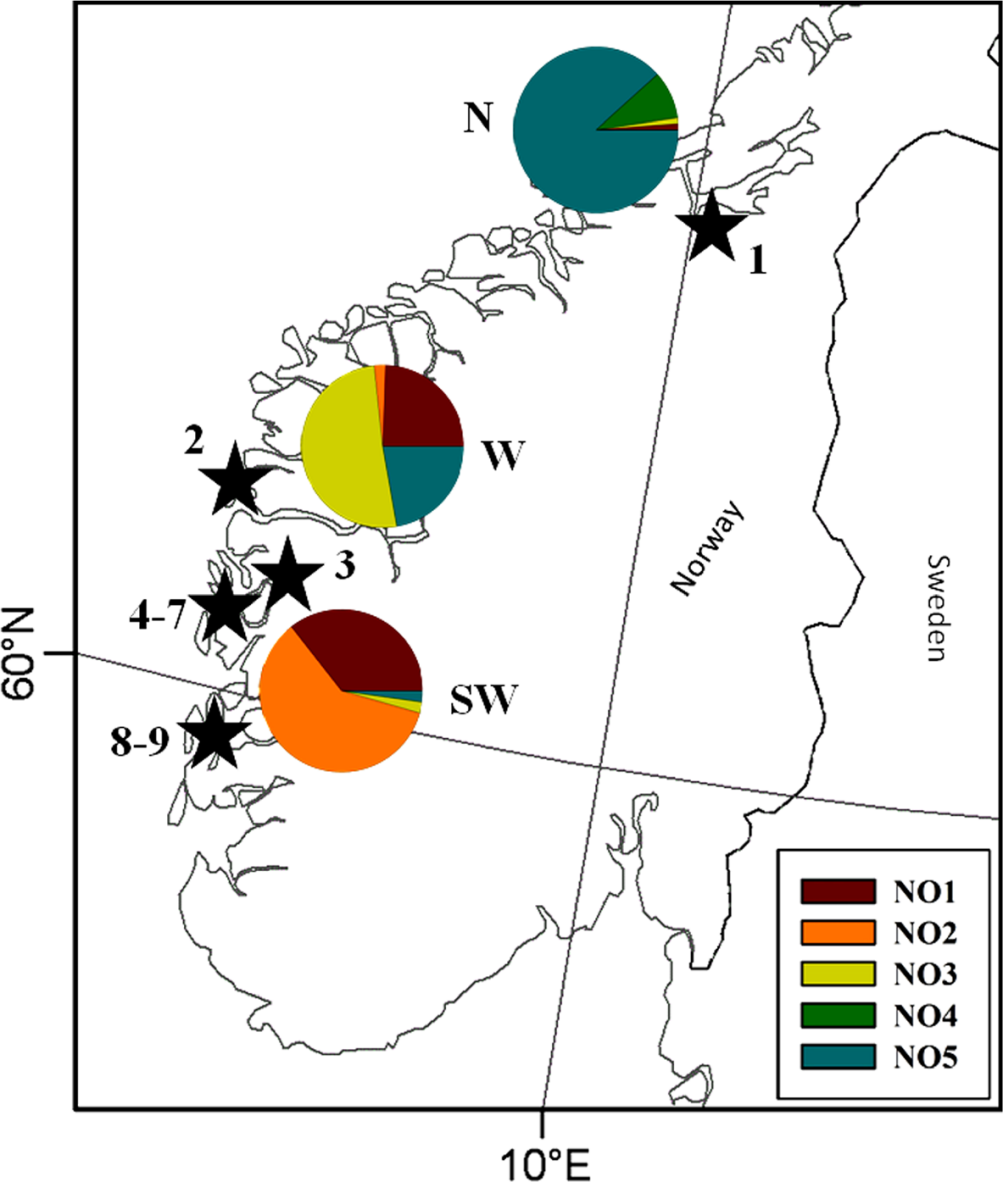
**Study sites and distribution of contemporary red deer mtDNA haplotypes in Norway.** Stars mark the distribution of archaeological locations with samples used in the aDNA analyses. 1-Erkebispegården, 2-Grønehelleren, 3-Skipshelleren, 4-Dreggsalmenningen, 5-Rosenkrantzgate, 6-Grimstadneset, 7-Ruskeneset, 8-Rundøyno, 9-Geitalemen. Pie charts show the relative distribution of haplotypes (NO1-NO5) in contemporary red deer in three geographic regions: SW, W and N.

A total of 142 Holocene samples of subfossil red deer skeletal remains were collected from nine archaeological sites in Norway for aDNA analysis of the mtDNA control region (D-loop) (Figure 1 and Table 1). Samples were ^14^C dated when originating from poorly dated sites or with an insecure stratigraphy (Additional file 1: Table S1). ^14^C dates were calibrated using CALIB 5.0.1 [21] with Incal04 calibration curve [22] and all dates reported in the text are in calendar years before present (yr BP). The age of the samples range from the Mesolithic to the Late Middle Ages (c. 7,000-500 yr BP) and all samples were collected from the University Museum of Bergen. Data on contemporary Norwegian and European mtDNA diversity were compiled from the literature ( [14,23-29], Haanes *et al*. unpublished results), excluding sequences from populations described by authors as translocated by humans during the last centuries. Nine new sequences from contemporary Norwegian animals were also included (see Additional file 1: Table S2 for overview of contemporary samples). 

**Table 1:** Archaeological sites from which samples were taken for aDNA analysis

**Site #**	**Site name**	**County**	**Municipality**	**c. Age (BP)**	**# sampl.**	**Success rate**
1	Erkebispegården	Sør-Trøndelag	Trondheim	500	5	60%
2	Grønehelleren	Sogn & Fjordane	Solund	4000-1400	21	48%
3	Skipshelleren	Hordaland	Vaksdal	7500-1800	68	51%
4	Dreggsalmenningen	Hordaland	Bergen	500	2	50%
5	Rosenkrantzgate	Hordaland	Bergen	500	20	95%
6	Grimstadneset	Hordaland	Bergen	2000	7	29%
7	Ruskeneset	Hordaland	Bergen	4000-2000	6	0%
8	Rundøyno	Hordaland	Bømlo	4000-2500	7	29%
9	Geitalemen	Hordaland	Sveio	3800	5	20%

### DNA extraction, amplification and sequencing

We drilled out 30–50 mg of powder from the samples using a Dremel multitool on slow speed. DNA was extracted using Qiagen DNeasy Tissue Kit by adding to the samples 300 μl Buffer ATL, 200 μl 1 M EDTA (pH 8) and 35 μl proteinase K, incubating in a thermomixer at 55°C over night. We used 450 μl of this in extractions following the Qiagen protocol. Polymerase chain reaction (PCR) amplifications of a 327 bp long sequence were performed using two pairs of overlapping primers which were first tested using contemporary samples of red deer:

I. (Ce116F) 5Â´-CCACCAACCACACAACAAAA-3Â´ with (Ce343R) 5Â´-GATCTAGGGGACGGGATACG-3Â´;

II. (CeCRF251) 5Â´-TGCCCCATGCATATAAGCATG-3Â´ with (CeCRR519) 5Â´-TAGGTGAGATGGCCCTGAAAAAAG-3Â´.

Amplifications were performed in 25 μl reaction mixtures containing 4–8 μl DNA extract, 0.625 U of *PfuTurbo* Hotstart DNA Polymerase (Stratagene), 2.5 μl 10x *Pfu* buffer, 12.5 pmol of each primer, 2.5 μg bovine serum albumin (Sigma) and 200 μM of each dNTP. The PCR profile was 2 min denaturation at 95°C followed by 46 cycles of 30 s denaturation at 95°C, 30 s annealing at 72°C, 60 s of extension at 72°C, and a final extension step of 10 min at 72°C. Amplified PCR products were cleaned using ExoSAP-IT (USB). Sequencing of both strands was performed using BigDye terminator cycle sequencing kit v.1.1 on an ABI 3100 genetic analyser. Sequences were inspected and aligned by eye with aid of MEGA v.4 [30].

### Authentication

Several precautions were taken to ensure amplification of authentic DNA from the ancient samples [31]. All equipment and working surfaces were cleaned using sodium hypochlorite, ethanol or UV-light. Drilling and extraction were done in designated labs physically separated from post-PCR laboratories, and where no previous work on red deer had been done before. Lab coats and breathing masks were used, gloves were changed frequently and drill bits were changed for each sample. Samples were mechanically cleaned and the outer surface was removed before drilling out the powder. Blank extraction and PCR controls were used in each reaction and only DNA sequences which could be replicated from at least two independent amplifications of each primer pair were used in the subsequent analyses. In addition, the primer pairs were overlapping for 84 bp of the target sequence yielding four replicates or more of the most variable region, thereby ensuring that nuclear copies (NUMTS) could be avoided [32]. We cloned and sequenced a sample of each new haplotype found, using 10 clones from each sample. Cloning was performed according to the Topo TA Cloning protocols (Invitrogen) with PCR-products extracted from gel using the MinElute Gel Extraction Kit protocol (Invitrogen).

### Analysing genetic diversity and population structure

A previous analysis using microsatellites found a strong genetic differentiation of the present Norwegian populations north and south of the Sognefjord area [33]. We therefore pooled our contemporary data into three geographic regions along a north–south transect: the northern part of the distribution, including Møre & Romsdal and Trøndelag counties (N); the Sognefjord area (W); and the southwest, including Rogaland and Hordaland counties (SW). We tested for population structure between the three areas by calculating F-statistics using only haplotype frequencies (F_ST_) [34] and Φ_ST_ which also accounts for genetic distance between haplotypes [35]. Significance values were obtained after 10,000 permutations using the software Arlequin v. 3.1 [36].

Estimates may become biased when analysing populations with a pronounced population structure [37-49], and to better meet assumptions of panmixia we excluded “site 1” from the ancient samples and the N region from the contemporary data set focusing only on “western Norway” on subsequent analyses of genetic diversity through time. The data was divided into four time periods which were treated as separate “populations” in the analyses: c. 7,000-3,500 yr BP (mid-Holocene); c. 2,500-2,000 yr BP (representing the period before any suspected bottleneck and translocations); c. 1,500-500 yr BP (a period including the human Migration Period, the Viking Age and the Middle Ages); and the present. We calculated standard genetic diversity indices, θ_k_[40], Tajima’s D [41] and Fu’s F_s_[42] on both the present and ancient data sets using the software Arlequin.

### Bayesian skyline plot

A Bayesian Skyline Plot (BSP) [43] implemented in BEAST v.1.6.1 [44] was used to explore past demographic changes in the red deer population. This approach was also used to obtain a direct estimate of substitution rate by using the age of individual sequences as calibrating information [45]. Sequence ages were rounded to the nearest 500 yr BP as most samples were not directly dated but were obtained from well dated stratigraphic layers with a certain age range (Additional file 1: Table S3). Substitution model was selected using the Aikake information criterion in jModelTest v. 0.1.1 [46,47], suggesting that the HKY model had the best fit to the data set [48]. Both a strict and a relaxed molecular clock were applied; the results were similar and a strict clock was used for the final runs. We summarized the lineage coalescent events into ten groups to smooth the estimates over time. MCMC was run for 100 mill iterations, sampling every 10,000th step and with a 10% burn-in. To estimate model parameters we ran two independent runs which were combined and inspected using TRACER v.1.5 [49]. To evaluate support for the BSP model we also did a run using a constant size coalescent tree prior instead of the BSP, comparing the different runs using Bayes factors [50].

### Approximate Bayesian computation analysis

**Figure 2:**
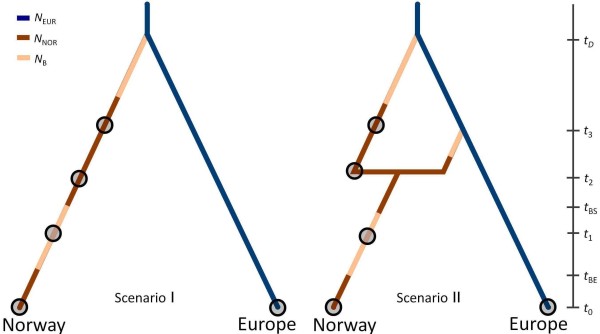
**Overview of the scenarios for the ABC analyses.** The scenarios depict one or two colonization events of red deer to Norway and estimated population sizes shown in different colours. t_0_ – t_3_ indicate DNA sampling points in time and are marked by circles. Note that we allow the sampling at t_2_ in scenario II to occur either before or after the admixture event. t_D_ denotes the divergence time of the Norwegian population, while t_BS_ and t_BE_ marks the start and end of the estimated population bottleneck respectively.

We estimated historical parameters for the Norwegian red deer; i.e. time since immigration(s), effective population size through time, and the genetic effect of bottleneck(s) in modern times; in a coalescence framework using approximate Bayesian computation (ABC) [51] and the DIY-ABC software ver. 1.0.4.37 [52]. Based on the results from the above analyses we explored two different scenarios with either one (scenario 1) or two colonisation events (scenario 2), with the second being a late translocation event possibly from the British Isles during the Viking ages (Figure 2). 

In two initial runs we compared the effects of using larger effective population size of females than of males (with a ratio of 0.1), compared to a scenario where they were equal, using similar parameter settings and prior ranges as described below. The results did not differ between the two approaches (results not shown), and we therefore did a full scale analysis assuming an equal sex ratio. We modelled two populations: Norway and Europe, with current effective population sizes *N*_NOR_ and *N*_EUR_ respectively. To represent the European diversity, one haplotype from 15 different western European populations were randomly picked, 13 from Skog *et al*. [14], one from Nielsen *et al*. [27] and one from McDevitt *et al*. [24]. Such sampling should yield properties approximating those of a random mating population [53,54]. We used the same time periods as described above to represent a serial sampling of the population at four time points (*t*_0_*-t*_3_), with *t*_0_ representing the contemporary sample and *t*_3_ the oldest of the ancient samples (Figure 2). In order to convert time estimates to years instead of generations, we assumed a generation time of four years in female red deer [25]. In scenario 1, the Norwegian population went through a population bottleneck starting at *t*_BS_ and ending at *t*_BE_ years back in time, where the effective population size of the Norwegian population is *N*_B_. At time *t*_1_, which we allow to occur either within or before the bottleneck period (i.e. we do not *a priori* determine the relative size of *t*_BS_ and *t*_1_), the Norwegian population is sampled, and prior range for *t*_1_ is set to between 500 and 1,700 yr BP. The sampling at year *t*_2_ and year *t*_3_ is assumed to happen before *t*_BS_, i.e., in a period with effective size *N*_NOR_, with prior ranges 2,000 – 2,500 yr BP for *t*_2_ and 3,500 – 7,000 yr BP for *t*_3_. In the period between *t*_D_ – *x* and *t*_D_ the Norwegian population experienced a founder event as a result of the colonisation of Norway, with *t*_D_ marking the time of divergence from the European population. In scenario 2 we assumed a similar history as in scenario 1, except that there was an immigration and admixture event from the European population before *t*_BS_ and either before or after the sampling at *t*_2_. The second immigrated population underwent a bottleneck for a total of *x* years and has an effective size *N*_NOR_ at the time of admixture. A fraction *r* of the original Norwegian population and a fraction 1 – *r* of the second immigration contribute to the admixed present day population.

We assumed no migration between populations after divergence, implying that the divergence time estimates are to be treated as minimum estimates. Prior ranges of the effective population size were set to 1,000 – 100,000 for *N*_NOR_ and *N*_B_, and 1,000 – 1,000,000 for *N*_EUR_. Based on the results from the Bayesian skyline plot (see below) we assumed that a putative bottleneck started between 500 – 2,500 yr BP and ended 1 – 500 years ago. The postglacial divergence of the Norwegian and European populations was set to occur between 9,000 and 20,000 yr BP. In scenario 2 the second divergence of the Norwegian population from the European was set to occur between 3,500 and 20,000 yr BP, with an original bottleneck lasting between 1 and 500 years. The admixture between the first and the second Norwegian populations occurred between 500 and 2,500 yr BP, before or after the sampling at *t*_2_.

Mutation rates were treated as nuisance parameters, and we assumed a Kimura 2-parameter model [55] with prior mutation rate estimate range obtained from the BEAST analyses (see below). Summary statistics were computed for each sample in each simulation, i.e. number of haplotypes, Tajima’s D, number of private segregating sites and average number of pairwise differences for each pair of populations sampled together. Each scenario was tested using 1 mill simulations, i.e., 2 mill simulations in total for the testing of scenarios 1 and 2.

The scenarios were compared using two approaches; one by directly comparing the summary statistics with the observed diversity in the data set and counting the frequency of the various scenarios among the most similar simulated datasets [56,57], and one by doing a logistic regression of each scenario probability for the most similar simulated data sets on the deviations between simulated and observed summary statistics [58,59]. In the direct comparisons approach the 500 simulated data closest to the observed values were used, while in the regression approach we used 1% of the simulated data closest to the observed data set. Confidence in scenario choice was evaluated by choosing scenario 1 as the true scenario and then simulating 500 data sets using this scenario and parameter values drawn randomly from the prior distributions, and then doing the same for scenario 2. The proportion of times the most likely scenario did not have the highest posterior probability when it was the true scenario was used as an estimate of type I error. The number of times the most likely scenario had the highest probability when it was not the true scenario was used as an estimate of type II error. Parameters were estimated for the most likely scenario using the 1% simulations for a given scenario most similar to the observed data set for the summary statistics employed. In order to evaluate the performance of the estimation procedure, we generated pseudo-observed data sets with known parameter values drawn from the posterior distribution given the most likely scenario. The mean relative bias (MRB), $\frac{1}{n}\sum_{i=1}^{n}\frac{e_{i}-v_{i}}{v_{i}}$, was estimated, where *e*_*i*_ is the *i*’th estimate of the pseudo-generated true value *v*, and averaged over the n = 500 data sets.

In order to study the impact of including aDNA samples, we chose the scenario with the highest estimated posterior probability (scenario 1, see below), and re-ran the ABC analyses using only extant DNA samples. In these simulations we assumed a similar history as in scenario 1 above, except that we had no aDNA samples, i.e. *t*_1_ – *t*_3_ were not included.

### Relationships between haplotypes

Relationships between haplotypes were investigated both on a national (Norwegian) and western European level using three different phylogenetic network methods: median-joining in Network v.4.6.0.0 [60], statistical parsimony in TCS v.1.21 [61] and NeighborNet in SplitsTree4 v.4.11.3 [62].

## Results

### Amplification success and authentication of ancient samples

**Figure 3:**
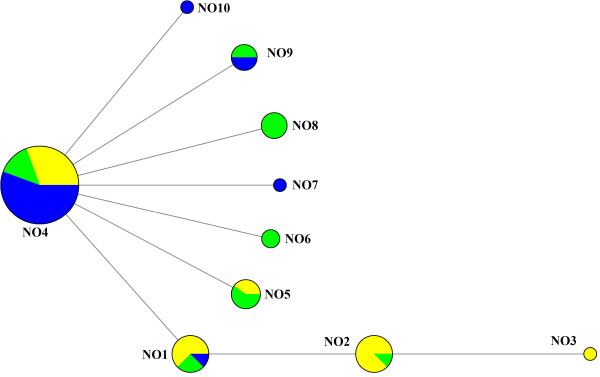
**Haplotype network of ancient Norwegian haplotypes.** Median-joining network of ancient Norwegian haplotypes showing their genetic relationship and frequency at different time periods. Yellow: bottleneck period (c. 500–1,500 yr BP), green: pre-bottleneck period (c. 2,000-2,500 yr BP), blue: Mid-Holocene (c. 3,500-7,000 yr BP). Size of the circles is scaled according to the frequency of sequences.

We successfully sequenced 73 (51.4%) of the ancient samples from which we identified 10 haplotypes: NO1 – NO10 [GenBank: JX861260-JX861269] (Table 1; Additional file 1: Table S3). A BLAST search [63] revealed that three haplotypes (NO8 – NO10) were novel. The three network methods yielded the same relationship between the ten Norwegian haplotypes showing a close relationship and a star-like pattern with NO4 as the central type (Figure 3). 

None of the negative controls contained red deer DNA, but some samples showed contamination from cattle (*Bos taurus*) when using primer pair II, a common problem in aDNA studies [64]. All clones produced identical sequences, except four clones in sample A1348 (NO9) which showed a G-A transition, typical of post-mortem DNA decay [65]. This was only expressed in the clones and was distinct from the segregating site that distinguished this haplotype. Most of the variable sites were also within the overlapping part of the two primer pairs and the independent replicates were always identical. We therefore have good reason to believe that all resulting sequences are authentic.

### Present population structure and diversity

176 control region sequences (n_N_ = 86; n_W_ = 45; n_SW_ = 45) were gathered from the extant Norwegian red deer distribution. From these we identified five different haplotypes (NO1 – NO5), identical to five of the ancient samples. The frequency and distribution of the haplotypes differed strongly among the three regions (Figure 1), with a significant genetic structure (global Φ_ST_ = 0.663, P < 0.001; global F_ST_ = 0.528, P < 0.001). Pairwise population differentiation was very high between regions and the most geographically distant populations were also the most genetically differentiated (Table 2). Overall haplotype diversity was moderate in the extant population while nucleotide diversity and θ_k_ were low, both seemingly lowest in the northernmost area (Table 2). Among the Tajima’s D or Fu’s F_s_ values only the D-value of the Sognefjord population (W) and the combined total genetic diversity of Norwegian deer differed significantly from equilibrium expectations, indicating a recent bottleneck.

**Table 2:** Present mtDNA genetic structure and diversity of red deer for three geographic regions in Norway

		**F-statistics**			**Genetic diversity indices**						
**Sample**	**# seq.**	**N**	**W**	**SW**	**h**	**s**	**H**_**d**_	**π**	**θ**_**k**_	**D**	**F**_**s**_
N	86	-	0.658	0.816	4	4	0.213	0.0009	0.707	−1.268	−1.680
W	45	0.507	-	0.204	4	4	0.643	0.0053	0.861	**2.063**	2.509
SW	45	0.656	0.339	-	4	4	0.524	0.0019	0.861	−0.763	−0.427
Total	176				5	4	0.687	0.0051	0.821	**2.537**	2.979

F-statistics: Φ_ST_ (above) and F_ST_ (below). Genetic diversity indices: number of haplotypes (h) and segregating sites (s), haplotype diversity (H_d_), nucleotide diversity (π), theta k (θ_k_). Significant values of Tajima’s D and Fu’s F_s_ in bold.

### Genetic diversity through time in western Norway

Considering only western Norway from where we have most of our ancient samples (70/73) it is clear that both the genetic diversity (Table 3) and the relative frequencies of haplotypes (Figure 3) have changed through time. The Early Iron Age samples (about 2,500-2,000 yr BP) show the highest diversity indices, while the number of haplotypes seems to have been reduced to the five types (NO1-NO5) found in the contemporary population already before the Middle Ages. Further loss of diversity is apparent when proceeding to the present as the once dominant haplotype NO4 has been lost in western Norway (W and SW areas) and is only found in the northern area in the present samples. θ_k_-values are higher in all the ancient periods compared to the present (Table 3), indicating larger effective female population size in the past. None of the Tajima’s D or Fu’s F_s_ values are significant except for the negative F_s_ value of the mid-Holocene samples (in bold), which remains significant even after Bonferroni correction, indicating population expansion. Among the five haplotypes found in the extant population all but NO3 have been found in samples older than any suspected translocation events.

**Table 3:** Genetic diversity through time in western Norway (W & SW areas)

**Sample**	**# seq.**	**h**	**s**	**H**_**d**_	**π**	**θ**_**k**_	**D**	**F**_**s**_
Present	90	4	4	0.735	0.0040	0.699	1.368	2.335
Ancient total	70	10	9	0.706	0.0034	2.962	−1.091	−3.756
c. 500–1500 yr BP	26	5	4	0.732	0.0036	1.558	0.355	−0.343
c. 2000–2500 yr BP	19	7	6	0.971	0.0043	3.543	−0.568	−2.420
c. 3500–7000 yr BP	25	5	4	0.363	0.0012	1.509	−1.699	**−3.414**

Notations as in Table 2.

### Bayesian skyline analysis

**Figure 4:**
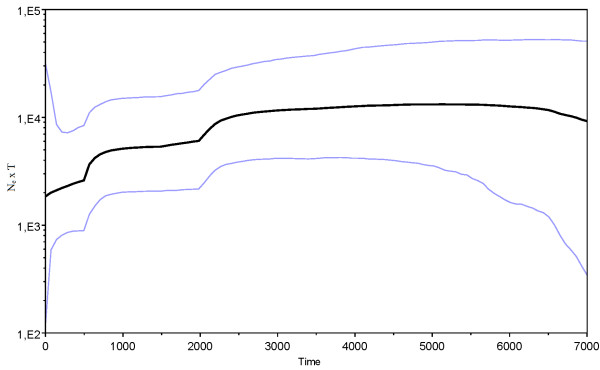
**Effective female population size through time.** Bayesian skyline plot (BSP) derived from the ancient and modern red deer dataset of western Norway. The x axis is in calendar years before present and y axis equals changes in effective population size (shown as the product of N_e_ and generation time T). Black line is the median estimate and the blue lines show the 95% highest posterior density intervals.

The BSP suggest a relatively stable female effective population size, although with a wide confidence interval, until about 2,000 yr BP, when effective size starts to decrease in a stepwise manner (Figure 4). A Bayes factor of 106.8 indicates a strong support for the BSP model over the constant size model. The estimated substitution rate was 2.78 ×10^-7^ (95% highest posterior density interval (HPDI): 8.15 ×10^-8^ – 5.23 ×10^-7^) substitutions per site per year, which is in line with other studies using aDNA datasets [66,67] and the rate estimated for red deer using only present European diversity [14]. 

### ABC analyses

Both in the direct and logistic approach, scenario 1 in Figure 2 has the highest support (Additional file 1: Figure S1), i.e. a single colonisation of Norway. The estimated type I and type II error rates are 13% and 21% respectively, implying a statistical power of 79%. The estimated historical parameters based on scenario 1 are presented in Table 4. Median effective sizes of female Norwegian and European populations are *N*_NOR_ = 7,160 (95% credible interval 1,870 – 32,000) and *N*_EUR_ = 1.03 mill (95% CI 325,000 – 3.2 mill) and the European and Norwegian populations seemingly diverged from one another *t*_D_ = 15,280 (95% CI 9,960 – 19,560) years ago. There are only weak signs of bottleneck as modal values of *N*_B_ = 10 (95% CI 1,110 – 85,700), but median value being *N*_B_ = 22,700, i.e., larger than the *N*_NOR_ estimate. The larger median value of *N*_B_ compared to *N*_NOR_ is likely an effect of assuming an equal population size before and after the bottleneck. The median time with varying population sizes is *t*_BS_ – *t*_BE_ = 976 years (95% CI 452 – 1,704). All time estimates have relatively flat posterior probability distributions and estimates must be interpreted with caution. The bias (MRB) is low (<1) for *N*_NOR_, *N*_EUR_ and *N*_B_ effective size estimates and the various time estimates, while moderate for estimated time with possible changes in effective sizes after divergence (*x*, MRB equals 1.60) (Table 4).

**Table 4:** ABC historical parameter values

**Parameter**	**Median**	**Mode**	**q**_**0.05**_	**q**_**0.95**_	**MRB**
*N*_NOR_	7160 (13 700)	3450 (7910)	1870 (4590)	32 000 (63 700)	0.11 (0.11)
*N*_EUR_	1 030 000 (1 000 000)	684 000 (639 000)	329 000 (325 000)	3 200 000 (2 530 000)	0.21 (0.17)
*N*_B_	22 700 (34 100)	10 (4530)	1110 (2540)	85 700 (92 500)	0.82 (5.17)
x	156 (252)	4 (4)	16 (24)	396 (476)	1.60 (1.61)
*t*_BE_	364 (252)	400 (4)	124 (28)	488 (476)	0.33 (2.24)
*t*_BS_	1340 (1492)	532 (1360)	576 (600)	2192 (2408)	0.17 (0.18)
*t*_D_	15 280 (15 000)	19 160 (18 520)	9960 (9760)	19 560 (19 560)	0.01 (0.05)
*t*_1_	1120	1268	560	1644	0.12
*t*_2_	2292	2452	2032	2480	<0.01
*t*_3_	4840	3552	3608	6720	0.05

Median, mode, quantiles and mean relative bias (MRB) for the posterior parameters calculated from scenario 1 including or excluding (in parentheses) ancient DNA as estimated from the ABC-analyses.

The results from the simulations only using extant samples of DNA, and assuming scenario 1, are shown in parentheses in Table 4. The precision of the estimates are markedly reduced for some of the parameters causing MRB estimates to increase up to 6.8 times for various parameters when excluding aDNA samples compared to estimates including aDNA (average increase in MRB equal to 3.14 across all comparable parameters). However, the parameter estimates have partly overlapping credible intervals and in general the estimates are very similar, except that credible intervals on average is 20% larger in analyses excluding aDNA, i.e., including aDNA is seemingly increasing the precision of estimates.

### Placement in the western European lineage

**Figure 5:**
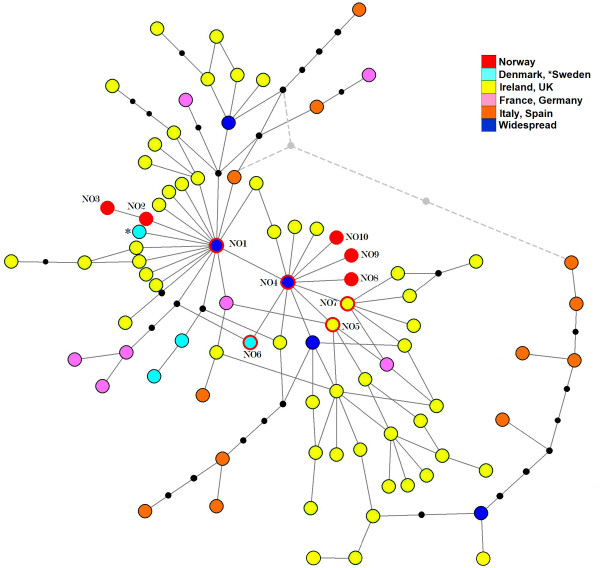
**Relationships between western European red deer.** Haplotype network of the western European lineage using both median-joining and statistical parsimony methods. Stapled line show connections only constructed when using the median-joining method. The branch lengths are not scaled to the number of substitutions and missing intermediate haplotypes are shown by black dots. Colour of circles and outlines indicates geographic location. Norwegian haplotypes are marked by named haplotypes. More info on haplotypes can be found in Additional file 1: Table S2.

We sampled 131 sequences from the western European lineage which resulted in 83 haplotypes of the analysed fragment (Additional file 1: Table S2). The median-joining and statistical parsimony methods produced similar haplotype networks with the exception of an additional loop in the median-joining (Figure 5). The haplotypes cluster in star-like patterns around two central haplotypes identical to NO1 and NO4, with the central haplotype considered to be NO1 by the TCS program. This dual clustering is also apparent in the NeighborNet (Additional file 1: Figure S2). Five of the Norwegian haplotypes (NO1, NO4 – NO7) are shared with other countries while the other five have so far only been found in Norway.

## Discussion

Red deer colonised western Norway some time before 8,000 yr BP and skeletal remains indicate that they were well established by the start of the period covered by this analysis (Rosvold *et al*. unpublished results). While significant Fu’s F_s_ indicate a population expansion during the early part of our study period (Table 3), the BSP (Figure 4) suggests a relatively stable female effective population size until about 2,000 yr BP. However, as the samples for this early period are widely dispersed in time, the posterior density interval of the BSP is large and the estimate is thus insecure. The Early Iron Age sample (c. 2,500 – 2,000 yr BP) has the highest diversity indices of all periods considered (Table 3), indicating that the population size may have been particularly high during this time.

Historical texts tell of a widespread and large population in Norway until around 500 yr BP, after which there was a dramatic decrease in numbers, allegedly caused by high harvesting rates and increased numbers of predators [18,68]. However, as indicated by the diversity indices, the BSP and the ABC analyses, this decrease appears to have been more prolonged, probably starting before medieval times. The estimated effective population sizes should be interpreted with caution [69,70], but the relative changes give a reliable picture of the magnitude of the demographic bottleneck. Accordingly, and supported by an earlier study on contemporary microsatellite DNA [11], the bottleneck seems not to have been as dramatic as the historic texts may suggest. The mtDNA diversity in the present population is, however, relatively low (Table 2) and especially the nucleotide diversity is low compared to other European populations [71]. The lowest diversity estimates are found in the northernmost region (N), as expected from a peripheral population loosing diversity during postglacial colonisation [72]. The current high population density of red deer in Norway is generally believed to be a recent phenomenon [17], but our results might indicate that at least the effective female population size, as measured through genetic diversity, was even higher in the past.

Following the spread of agriculture along the coast of western Norway the once dense coastal forests were transformed into the present day heathlands [73,74]. A process which was greatly accelerated from around 2,000 yr BP through intensified agricultural activities [75], iron, coal, and salt production, and later by mining and timber export [76]. These changes in the landscape probably facilitated and exacerbated the effects of heavy hunting by increasing habitat fragmentation and possibly reducing migration between areas, thereby isolating populations. The loss of the previously most abundant haplotype (NO4) may indicate extensive genetic drift within these fragmented populations, reducing genetic diversity on a local scale. However, the number of isolated populations were relatively large (at least six) and evenly spread along large parts of its former distribution [20]. Thus, the overall genetic diversity may have been better maintained by the wide geographic spread of the populations than if they had been reduced to a single but larger population [77]. Population fragmentation and isolation is expected to cause increased genetic differentiation [2], and indeed, there is a high degree of genetic structuring among the present Norwegian female red deer (Figure 1 and Table 2). This indicates that few females have migrated between the areas since the population size reduction, supporting findings that fjords may act as significant dispersal barriers [34,78].

The ten Norwegian haplotypes observed in the ancient samples are closely related to the rest of the western European clade. The star-like structuring (Figure 3) coupled with low nucleotide diversity (Table 3) is indicative of a population expansion from an ancestral haplotype [79] which in this case seems to be NO4. This close relationship, coupled with the observation that all present-day haplotypes except NO3 have been found in samples dating to 2,000 yr BP or older, is an indication of no human translocation of female red deer into Norway during historic times. This conclusion is also supported by ABC analyses, where a scenario of only one post-glacial colonisation of Norway gets highest support. NO3 is first found at low frequency in late medieval samples and has so far only been found in Norway (Figure 5). It is possible that NO3 originated in Norway and became frequent in the Sognefjord area (W, Figure 1) as an effect of genetic drift and subsequent population increase during recent times.

An estimated divergence time of the Norwegian population of around 15,000 yr BP (9,960 – 19,560), as indicated by the ABC analysis (Table 4), coincides with the start of the northward colonisation of Europe after the Last Glacial Maximum [9]. The haplotype network for the western European red deer (Figure 5) confirms previous findings of a close relationship within the western European clade [13,14], with little or no apparent geographic structure and several cross-links indicating uncertain relationships. Out of the ten haplotypes found in the ancient Norwegian dataset five are shared with other countries. Of these, the two central haplotypes NO1 and NO4 are widespread, being present in Scotland and the border forests between Germany and the Czech Republic [14,23,25], with NO4 also found in Spain [14], and NO1 being one of the most common types found in the Scottish highlands today [25]. None of the extant Norwegian haplotypes are shared with other Scandinavian countries; although the ancient NO6 is found in Denmark today [27]. The Swedish population seems to have experienced a more severe bottleneck than the Norwegian as only one haplotype, closely related to NO1, is found among indigenous animals [11,14]. This low diversity makes it hard to postulate the relationship to Swedish animals, but present Scandinavian diversity indicates that some haplotypes never reached Norway and that a large part of those passing through Denmark during the post-glacial colonization (i.e. the Norwegian types) were later lost. Sampling aDNA from both Sweden and Denmark could shed more light on if this was caused by genetic bottlenecks or if they were replaced by later immigrants that never reached Norway.

Two star shaped patterns are apparent among the western European samples, separated by an A-G transition. One of these centres on NO1, which has been described before [14], while the other centres on the closely related NO4 and is made more apparent by our ancient samples. This could be an indication of two subgroups within the western European haplogroup, possibly reflecting different refugial areas in France and Iberia [9]. Most of the European populations have undergone severe population reductions during recent centuries and several translocations which could have distorted any phylogeographic patterns within the haplogroup [6,71]. A large-scale sampling of aDNA from other European populations would thus provide valuable insights into the phylogeography of European red deer.

## Conclusions

Our results indicate that the current genetic diversity of Norwegian red deer can be explained by one post-glacial immigration event followed by a gradual loss of diversity and increasing population sub-division due to heavy exploitation and habitat fragmentation during the last two millennia. They also show that only females from the western European red deer lineage colonised Scandinavia, supporting previous findings that the eastern lineage had a more limited dispersal into Europe [14,80]. The population decline seems to have been more prolonged in time than what is reflected in historic documents, indicating that even early human land use practices had an effect on red deer.

## Competing interests

The authors declare that they have no competing interests.

## Authors’ contributions

JR did the laboratory work, analysed the data and drafted the manuscript. HKS performed the ABC-analyses. JR and AKH picked out the ancient samples. KHR supervised the laboratory work. All authors were involved in the study design, revising of the manuscript and read and approved the final manuscript.

## Supplementary Material

Additional file 1 **Additional file contains details of data collection and some aspects of data analyses.** It also contains information on dating of ancient samples as well as an overview of the new sequences provided by this study. The file is in PDF format. Click here for file
