# Supplementary material for: Reconstructing the history of a fragmented and heavily exploited red deer population using ancient and contemporary DNA
Source: BMC Evol Biol. 2012 Sep 26;12:191. doi: 10.1186/1471-2148-12-191 (PMC3514237; doi:10.1186/1471-2148-12-191)
Supplement: Additional file 1 — Additional file contains details of data collection and some aspects of data analyses. It also contains information on dating of ancient samples as well as an overview of the new sequences provided by this study. The file is in PDF format. [file 1471-2148-12-191-S1.pdf]

Additional file 1

Figures

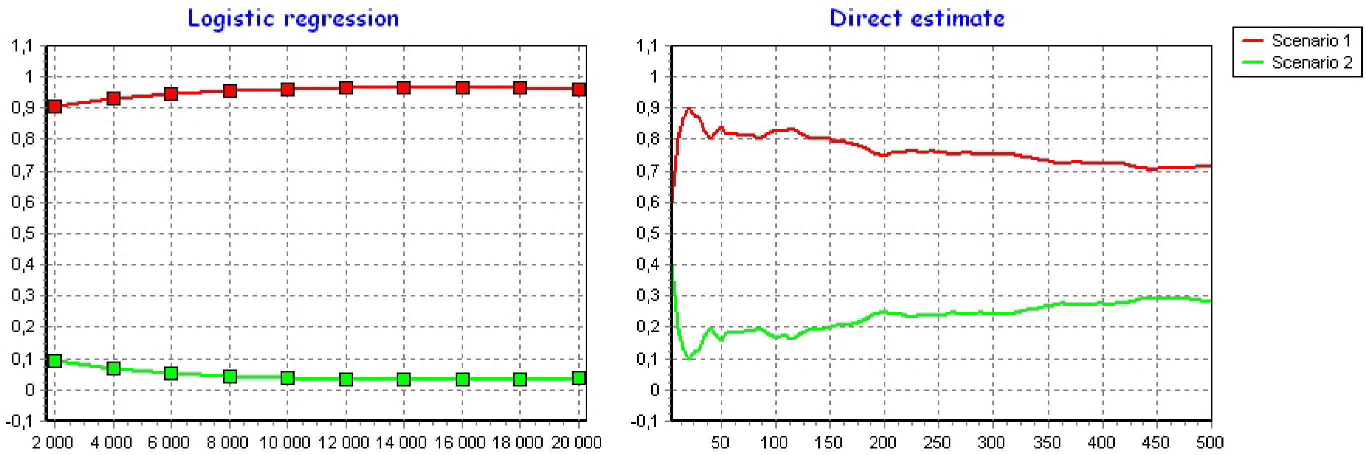

Figure S1 Confidence in scenario choice. Posterior probabilities of the two scenarios using logistic regression and direct comparison approach.

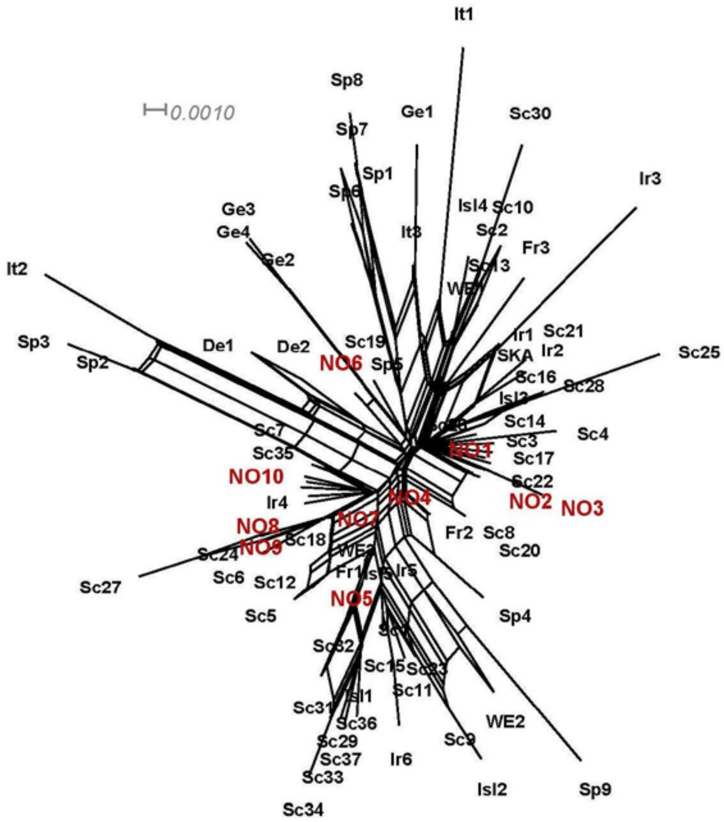

Figure S2 Haplotype network of red deer from the western European lineage constructed using the NeighborNet method implemented in SplitsTree4 v.4.11.3. Norwegian haplotypes highlighted in red. Notations refer to haplotypes in Table S2.

Tables

Table S1 New radiocarbon dates on samples from insecurely dated sites

Sample ID	Site	Age	Lab. ref
1305	Geitalemen	3555 ± 30	TRa-70
1311	Rundøyno	2480 ± 25	TRa-68
1316	Rundøyno	3615 ± 30	TRa-69
1322	Grimstadneset	2040 ± 55	TRa-66
1324	Grimstadneset	1940 ± 30	TRa-67
1332	Grønehelleren	1760 ± 30	TRa-72
1333	Grønehelleren	1650 ± 40	TRa-3066
1335	Grønehelleren	1470 ± 40	TRa-3067
1336	Grønehelleren	4065 ± 45	TRa-3068
1340	Grønehelleren	1560 ± 25	TRa-73
1342	Grønehelleren	1570 ± 40	TRa-3069
1343	Grønehelleren	3080 ± 40	TRa-3070
1344	Grønehelleren	3620 ± 45	TRa-3071
1345	Grønehelleren	1675 ± 40	TRa-3072
1348	Grønehelleren	3120 ± 40	TRa-3073

Table S2 References to red deer haplotypes from the western European lineage used to construct haplotype networks

Haplotype information		Sampling references*							
Haplotype	Countries present	This study	1	2	3	4	5	6	7
NO1	Norway, Scotland, Czech Rep.	NO1	AC1, AD5		H2, H5, H8, H20, H32, H35, H48, H50, H59, H61, H62			HT02	HT10
NO2	Norway	NO2	AC3						
NO3	Norway	NO3	AC4						
NO4	Norway, Scotland, Spain, Germany, Czech Rep.	NO4	AA1, AD6, AD7		H4, H6, H63, H64			HT01	HT8
NO5	Norway, Scotland	NO5	AA7	AF291888	H56				HT7
NO6	Norway, Denmark	NO6				H1, H5			
NO7	Norway, Scotland	NO7			H39				
NO8	Norway	NO8							
NO9	Norway	NO9							
NO10	Norway	NO10							
SKA	Sweden, Denmark		AC2			H3			
De1	Denmark					H6			
De2	Denmark					H7			
Fr1	France		AA8						
Fr2	France		AA9						
Fr3	France		AD2						
Ge1	Germany		AB3						
Ge2	Germany		AC5						
Ge3	Germany		AC6						
Ge4	Germany		AC7						
Isl1	Scotland, Ireland		AD8		H19, H68, H74		MY1		HT9

Isl2	Scotland, Ireland	AD9	H24, H25, H53		NW2	HT6
Isl3	Scotland, Ireland		H1, H3, H15, H29, H40		KNP1, KNP4	HT2
Isl4	Scotland, Ireland, England		H16		NW1	HT4
Isl5	Scotland, Ireland		H65		GY3	
WE1	Denmark, France, Scotland, Ireland	AD1	H9, H60	H2	NW3	
WE2	France, Scotland, Ireland, England	AA3	H18		GY2	HT3
WE3	Spain, Scotland	AA2	H38			
Ir1	Ireland				KNP2, KNP5, KNP7	
Ir2	Ireland				KNP3	
Ir3	Ireland				KNP6	
Ir4	Ireland				GY1	
Ir5	Ireland				GY4	
Ir6	Ireland				WK2	
Sc1	Scotland	AD10	H58			HT13
Sc2	Scotland	AD4	H66			HT11
Sc3	Scotland		H7			
Sc4	Scotland		H10			
Sc5	Scotland		H11			
Sc6	Scotland		H12			
Sc7	Scotland		H13			
Sc8	Scotland		H14			
Sc9	Scotland		H17			
Sc10	Scotland		H21			
Sc11	Scotland		H22			
Sc12	Scotland		H23			
Sc13	Scotland		H26			
Sc14	Scotland		H27			
Sc15	Scotland		H28			
Sc16	Scotland		H30			
Sc17	Scotland		H31			
Sc18	Scotland		H33			
Sc19	Scotland		H34			
Sc20	Scotland		H36, H49			
Sc21	Scotland		H37			
Sc22	Scotland		H41			
Sc23	Scotland		H42			
Sc24	Scotland		H43			
Sc25	Scotland		H44			
Sc26	Scotland		H45			
Sc27	Scotland		H46			
Sc28	Scotland		H47			
Sc29	Scotland		H52			
Sc30	Scotland		H54			
Sc31	Scotland		H55			

Sc32	Scotland			H57				
Sc33	Scotland			H67, H73				
Sc34	Scotland			H69				
Sc35	Scotland			H70				
Sc36	Scotland			H71				
Sc37	Scotland			H72				
Sc38	Scotland							HT5
Sc39	Scotland							HT12
Sp1	Spain		AF291889					
Sp2	Spain	AA4						
Sp3	Spain	AA5						
Sp4	Spain	AA10						
Sp5	Spain	AB1						
Sp6	Spain	AB4						
Sp7	Spain	AB5						
Sp8	Spain	AB6						
Sp9	Spain	AB7						
It1	Italy	AD3	AF291887					
It2	Italy	AA6						
It3	Italy	AB2						

* Haplotype name given by the respective authors

Table S3 Haplotypes of ancient Norwegian samples and modern samples from primer testing, with age used in BEAST analyses and reference to the dating of the site

Sample #	Site	Dating method	BEAST age*	NO Haplotype	Reference
A1194	Skipshelleren	dated layer	2000	4	8
A1197	Skipshelleren	dated layer	2000	5	8
A1199	Skipshelleren	dated layer	2000	4	8
A1201	Skipshelleren	dated layer	2000	4	8
A1202	Skipshelleren	dated layer	2000	9	8
A1208	Skipshelleren	dated layer	2000	8	8
A1209	Skipshelleren	dated layer	2000	6	8
A1211	Skipshelleren	dated layer	2000	8	8
A1212	Skipshelleren	dated layer	2000	8	8
A1213	Skipshelleren	dated layer	2000	8	8
A1214	Skipshelleren	dated layer	2000	5	8
A1215	Skipshelleren	dated layer	2000	1	8
A1216	Skipshelleren	dated layer	2000	4	8
A1218	Skipshelleren	dated layer	2000	9	8
A1222	Skipshelleren	dated layer	2000	6	8
A1223	Skipshelleren	dated layer	2000	1	8
A1229	Skipshelleren	dated layer	5500	4	8
A1236	Skipshelleren	dated layer	6500	4	8
A1237	Skipshelleren	dated layer	6500	4	8
A1238	Skipshelleren	dated layer	7000	4	8
A1239	Skipshelleren	dated layer	4500	4	8
A1292	Erkebispegården	dated site	NA	3	9
A1294	Erkebispegården	dated site	NA	5	9

A1295	Erkebispegården	dated site	NA	5	9
A1296	Dreggsalmenningen	dated site	500	2	10
A1305	Geitalemen	dated sample	4000	4	Supl. 1
A1311	Rundøyno	dated sample	2500	2	Supl. 1
A1316	Rundøyno	dated sample	4000	10	Supl. 1
A1322	Grimstadneset	dated sample	2000	4	Supl. 1
A1324	Grimstadneset	dated sample	2000	5	Supl. 1
A1332	Grønehelleren	dated sample	1500	2	Supl. 1
A1333	Grønehelleren	dated sample	1500	5	Supl. 1
A1335	Grønehelleren	dated sample	1500	2	Supl. 1
A1340	Grønehelleren	dated sample	1500	1	Supl. 1
A1342	Grønehelleren	dated sample	1500	2	Supl. 1
A1343	Grønehelleren	dated sample	3500	9	Supl. 1
A1344	Grønehelleren	dated sample	4000	4	Supl. 1
A1345	Grønehelleren	dated sample	1500	4	Supl. 1
A1346	Grønehelleren	dated layer	3500	4	Supl. 1
A1348	Grønehelleren	dated sample	3500	9	Supl. 1
A1939	Skipshelleren	dated layer	5500	4	8
A1942	Skipshelleren	dated layer	5000	4	8
A1943	Skipshelleren	dated layer	4500	4	8
A1944	Skipshelleren	dated layer	5500	4	8
A1945	Skipshelleren	dated layer	4500	4	8
A1946	Skipshelleren	dated layer	5500	7	8
A1948	Skipshelleren	dated layer	5000	4	8
A1949	Skipshelleren	dated layer	7000	4	8
A1950	Skipshelleren	dated layer	6500	1	8
A1952	Skipshelleren	dated layer	5500	4	8
A1954	Skipshelleren	dated layer	4500	4	8
A1956	Skipshelleren	dated layer	6500	4	8
A1957	Skipshelleren	dated layer	7000	4	8
A1959	Skipshelleren	dated layer	7000	4	8
A2054	Rosenkrantzgate	dated site	500	4	11
A2055	Rosenkrantzgate	dated site	500	4	11
A2056	Rosenkrantzgate	dated site	500	4	11
A2057	Rosenkrantzgate	dated site	500	2	11
A2059	Rosenkrantzgate	dated site	500	4	11
A2060	Rosenkrantzgate	dated site	500	1	11
A2061	Rosenkrantzgate	dated site	500	4	11
A2062	Rosenkrantzgate	dated site	500	3	11
A2063	Rosenkrantzgate	dated site	500	4	11
A2064	Rosenkrantzgate	dated site	500	2	11
A2065	Rosenkrantzgate	dated site	500	4	11
A2066	Rosenkrantzgate	dated site	500	1	11
A2067	Rosenkrantzgate	dated site	500	1	11
A2068	Rosenkrantzgate	dated site	500	5	11
A2069	Rosenkrantzgate	dated site	500	4	11
A2070	Rosenkrantzgate	dated site	500	4	11
A2071	Rosenkrantzgate	dated site	500	2	11
A2072	Rosenkrantzgate	dated site	500	4	11
A2073	Rosenkrantzgate	dated site	500	1	11
M1422	Hitra	contemporary	0	4	
M1690	Mongstad	contemporary	0	1	
M1691	Mongstad	contemporary	0	1	
M1692	Mongstad	contemporary	0	3	

M1693	Mongstad	contemporary	0	1
M1727	Ølve	contemporary	0	2
M1728	Ølve	contemporary	0	1
M1729	Ølve	contemporary	0	2
M1730	Ølve	contemporary	0	2

* Age used in the Bayesian skyline plot analysis

References:

1: Skog, A., F. E. Zachos, et al. (2009). "Phylogeography of red deer (*Cervus elaphus*) in Europe." Journal of Biogeography 36: 66-77

2: Randi, E., N. Mucci, et al. (2001). "A mitochondrial DNA control region phylogeny of the Cervinae: speciation in Cervus and implications for conservation." Animal Conservation 4: 1-11

3: Pérez-Espona, S., F. J. Pérez-Barberia, et al. (2009). "Genetic diversity and population structure of Scottish Highland red deer (*Cervus elaphus*) populations: a mitochondrial survey." Heredity 102: 199-210

4: Nielsen, E. K., C. R. Olesen, et al. (2008). "Genetic structure of the Danish red deer (*Cervus elaphus*)." Biological Journal of the Linnean Society 95: 688-701

5: McDevitt, A. D., C. J. Edwards, et al. (2009). "Genetic structure of, and hybridisation between, red (*Cervus elaphus*) and sika (*Cervus nippon*) deer in Ireland." Mammalian Biology 74: 263-273

6: Fickel, J., Bubliy, O.A., et al. (2012). “Crossing the border? Structure of the red deer (*Cervus elaphus*) population from the Bavarian–Bohemian forest ecosystem.” Mammalian Biology 77: 211-220

7: Hmwe, S. S., Zachos, F. E., et al. (2006). “Genetic variability and differentiation in red deer (*Cervus elaphus*) from Scotland and England.” Journal of Zoology 270: 479-487

8: Rosvold, J. (2006). Do single prehistoric dwelling sites reveal changes in the relative abundance of moose to red deer in western Norway? Skipshelleren and the Viste cave. Department of Biology. Trondheim, NTNU. MSc thesis

9: Hufthammer, A. K. (1999). "Utgravningene i Erkebispegården i Trondheim. Kosthold og erverv i Erkebispegården." NIKU Temahefte 17: 1-47

10: Undheim, P. (1985). Osteologisk materiale fra Dreggen: en økologisk studie fra middelalderens Bergen. Bergen Museum. Bergen, University of Bergen. Cand. Philol. Thesis

11: Wiig, Ø. (1981). "Faunal remains from Mediaeval Bergen." Fauna Norvegica Series A 2: 34-40
